# Investigating the status of marital burnout and related factors in married women referred to health centers

**DOI:** 10.1186/s12905-021-01172-0

**Published:** 2021-01-13

**Authors:** Mahbobeh Nejatian, Ali Alami, Vahideh Momeniyan, Ali Delshad Noghabi, Alireza Jafari

**Affiliations:** 1 Social Determinants of Health Research Center, Gonabad University of Medical Sciences, Gonabad, Iran; 2 Department of Epidemiology and Biostatistics, School of Public Health; Social Determinants of Health Research Center, Gonabad University of Medical Sciences, Gonabad, Iran; 3 Department of Psychology, Ferdowsi University of Mashhad, Mashhad, Iran

**Keywords:** Women's health, Marital burnout, Women burnout, Mental health, Marital boredom

## Abstract

**Background:**

Marital burnout is an important issue in marriage and many factors play an important role in this phenomenon. The aim of this study was to determine the status of marital burnout and the factors affecting married women who were referred to health centers because of it.

**Methods:**

In this study, 936 women were selected by multistage sampling and data collection was performed using questionnaires of demographic and couple burnout. Data analysis was performed using SPSS software version 24.

**Results:**

The mean (± SD) of marital burnout, in this study, was 55.46 (± 18.03) (out of 147 score). There was a significant relationship between the level of women's education with total marital burnout, and the subscales of somatic and emotional burnout (*P* < 0.05). A significant relationship was also observed between mandatory marriage and total marital burnout, as well as subscales of somatic, emotional, and psychological burnout (*P* < 0.05). A significant relationship was detected and observed between women's participation in training courses of communication skills and total marital burnout, inclusive of the subscales regarding psychological burnout (*P* < 0.05). The results of linear regression showed a significant relationship between mandatory in marriage, marital satisfaction, marriage duration, and husband's level of education with women's marital burnout. The variables were finally able to predict 12% of marital burnout variance. It should be noted that marital satisfaction had a higher effect on predicting marital burnout (*P* < 0.001).

**Conclusions:**

Marital satisfaction was one of the effective factors in predicting marital burnout, so it can be concluded that it is necessary to pay more attention to this issue. Educational programs and examining the factors that enhance marital satisfaction are needed to prevent and reduce marital burnout in married couples.

## Background

Burnout was originally used to help professions and defined as “the experience of exhaustion, where the people who suffer from it become cynical toward the value of their occupation and doubt their ability to perform” [1], but nowadays it can affect everybody and is used by everyone from celebrities to housewives [2]. A stressful lifestyle can put people under a lot of stress, to the point that they feel empty, tired, burning, and unable to cope [2, 3]. Burnout includes three dimensions of emotional, physical, and psychological and various factors affect it [4, 5].

One of the problems that couples may experience in their married life is marital burnout, therefore, the couple should take the necessary measures to prevent and reduce this excessive stress [6, 7]. Marital burnout, caused by long-term conflicts between couples, increases aggressive behavior and reduces love they may once had for each other, which ultimately reduces the quality of married life and dissatisfaction wedges in between them [8].

Pines believes that when the magic of romantic love disappears over time, their lives lose the meaning they thought their lover would give them [9]. Marital burnout is one of the main reasons for marital disputes and the lack of intimacy between them. The most severe form of marital burnout leads to the breakdown of marital relationships and divorce. Marital burnout is also related to how couples communicate, resolve conflicts, and problem-solving skills, and couples who are more proficient in these areas experience less burnout [10].

Ferri et al. believe that burnout occurs when coping strategies to overcome stress become ineffective, resulting in a person suffering from chronic physical and mental illness. Feeling tired, lethargic, anorexic, chronic headaches, abdominal pain, and overeating are some of the causes of somatic burnout [11]. Feelings of hopelessness, sadness, feelings of emptiness, meaninglessness, and depression are among the symptoms of mental burnout. Decreased self-esteem, negative emotions, and frustration with one's spouse are among the symptoms of emotional burnout [11].

Based on the results of a study low marital burnout and increased quality of life reduced mortality in married individuals [12]. There is a significant relationship between marital dissatisfaction and depression. Increasing marital dissatisfaction among couples in turn increases the risk of depression [13]. When symptoms of depression increase among couples it also strengthens other stressors, ultimately reducing the quality of marital harmony between them [13, 14]. Burnout can also cause physical problems such as musculoskeletal tension and cardiovascular disease in those experiencing the burnout [15].

In the majority of findings from studies of couples who suffer from marital burnout, it has been reported that women experience more burnout than men [5, 16, 17]. The reason for the higher levels of burnout in women is that women start their marriage with higher expectations than men [18]. Problems and stress that married women endure when trying to perform their duties as spouse or mother is more than the stress that men experience as a spouse or father [18].

Women have more roles to play in married life than men do. Traditionally, most housework chores are done by married women, and in addition to housework, they may also have a job outside their home. Women with multiple roles may suffer from more stress and strain due to the high pressure of their responsibilities and the lack of leisure time [19–22]. The results of a study showed that there is a relationship between a husband’s involvement in housework and his wife’s psychosocial health, which in turn helps improve her psychosocial health [23]. In this context, the aim of the present study was to investigate marital burnout and determine the factors effecting married women who had been referred to various health centers in our area. The research hypothesis was that the demographic factors affect the marital burnout among married women.

## Methods

This cross-sectional analytical study was conducted to investigate the marital burnout of married women with a sample size of 936 people in Gonabad, Iran. Based on the previous study [24] and with a 95% confidence level, test power of 0.80, the accuracy of 0.66, and standard deviation of 5.33, the sample size was estimated through the following formula of 936 people.

$$n=\frac{{({z}_{1-\frac{\alpha }{2}})}^{2} {(s)}^{2} }{{(d)}^{2}}$$

### Sampling method

In this study, subjects were selected by multistage sampling. In the first stage, the stratified sampling method was used according to the target population. For this purpose, first, the list of all health centers was determined and each center was considered as one category. In proportion to the population of each category, the samples were randomly selected from women referring to health centers that had inclusion criteria. In this study, inclusion criteria include at least one year has passed since the person's marriage, currently living with a spouse, and a willingness to participate in the study.

### Data collection tools

In this study, data collection was performed using questionnaires of demographic and marital burnout scale.

#### Demographic questionnaire

This questionnaire includes questions such as age, duration of marriage, level of education, occupation, and number of children.

#### Marital burnout scale

To measure the degree of marital burnout of married women, the Pines marital burnout scale was used. This scale consists of 21 questions and three subscales; somatic burnout (being tired, being physically exhausted, being wiped out, feeling rundown, being weary, feeling weak, feeling energetic), emotional burnout (feeling depressed, being emotionally exhausted, feeling burned out, feeling worthless, being troubled, feeling hopeless, feeling anxious), and psychological burnout (having a good day, being happy, feeling unhappy, feeling trapped, feeling disillusioned and resentful about people, feeling rejected, and feeling optimistic).

Each of the subscales consists of 7 questions that are measured with a 7-point Likert scale (never, once in a long time, rarely, sometimes, usually, often, always). The lowest score on each scale is 7 and the highest score is 49. To obtain the total burnout rate, the scores of each subscale obtained were added together and the mean score of the individual burnout was calculated. Finally, the mean score of the individual was calculated between 21 and 147. A higher score indicated a higher level of marital burnout [25, 26]. The validity and reliability of this questionnaire has been investigated in Iran by Ahmadi et al., and its Cronbach's alpha rate was reported to be 0.82 [27].

### Data analysis

Data analysis was performed using SPSS software version 24. To describe the data, the frequency and percentage or mean and standard deviation were used. The Independent Samples T-test and One-way ANOVA were used to compare the mean of variables in binary and categorical variables, respectively. Pearson correlation Coefficient was used to assess the correlation between the (total marital burnout, subscales of somatic, emotional, and psychological burnout). Also, a linear regression test was used to investigate the predictor factors for (marital burnout). Significant levels for data analysis were considered less than 0.05.

### Ethical considerations

The study process began with the approval of the Vice Chancellor for Research. First, the objectives of the research project were explained to the subjects, and after obtaining written informed consent from the people, the questionnaires were completed by self-report. Individuals were also assured that their information would remain confidential to the research team.

## Results

According to the results of this study, the mean (± SD) of the participants was 35.95 (± 10.23) and most women (36.7%) were between 31 and 40 years old. 74.6% of the housewives were employed and 60.1% of their husbands were self- employed. Most of the women in the study had a high school education, and most of their husbands had a college education. In this study, 42.5% of the participants reported that they had known their spouse before marriage. Only 22.4% reported that they had participated in communication skills training with their spouse. Other demographic information can be seen in Table 1.

**Table 1:** Frequency distribution of demographic variables

Variable		n	%	Variable		n	%
Age	17–30	305	32.7	Husband's age	17–30	179	19.4
	31–40	342	36.7		31–40	314	34
	41–50	204	21.9		41–50	275	29.8
	51 <	81	8.7		51 <	156	16.9
Occupation	Unemployed	691	74.6	Husband's Occupation	Employee	370	39.9
	Employee	235	25.4		Self- Employee	558	60.1
Level of education	Illiterate	10	1.1	Husband's education	Illiterate	16	1.7
	elementary	139	14.9		elementary	74	8
	middle school	122	13.1		middle school	172	18.5
	High school	382	41.1		High school	294	31.7
	Academic	277	29.8		Academic	372	40.1
Number of children	does not have	15	1.8	Relative to spouse	Relative	329	35.4
	One	169	20.7		Non-relative	559	64.4
	Two	337	41.3	Meet the wife before marriage	Yes	397	42.5
	Three or more	294	36.1		No	538	57.5
	missing	125					
Duration of marriage	1–10	288	31.7	Marital satisfaction	Yes	882	94.5
	11–20	301	33.1		No	51	5.5
	21–30	199	21.9	Participate in communication skills training courses	Yes	209	22.4
	31–40	99	10.9		No	723	77.6
	41 <	21	2.3	Mandatory marriage	Yes	49	5.2
					No	887	94.8

In this study, the mean (± SD) of marital burnout was 55.46 (± 18.03). The mean (± SD) subscales of marital burnout can be seen in Table 2. The results of the One-way ANOVA test showed that there was a significant relationship between women's age with total marital burnout and subscale of somatic burnout. Also, there was a significant relationship between the husband's age with total marital burnout and the subscales of somatic and psychological burnout in women (*P* < 0.05). The results of the One-way ANOVA test showed that there was a significant relationship between the duration of marriage with total marital burnout and the subscale of somatic burnout (*P* < 0.05).

**Table 2:** Mean (SD) marital burnout and its subscales

Variable	n	Mean	SD	Score range	Alpha Cronbach's
Somatic	936	19.43	6.72	7–49	0.72
Emotional	936	18.49	7.65	7–49	0.83
Psychological	936	17.53	5.88	7–49	0.72
Total marital burnout	936	55.46	18.03	21–147	0.90

According to the results of the Independent Samples T-test used in this study, there was a significant relationship between their job and married women’s total marital burnout, and the subscale of emotional and psychological burnout in these women (*P* < 0.05). The results of the One-way ANOVA test also showed that there was a significant relationship between the level of education in women with total marital burnout and the subscales of somatic and emotional burnout (*P* < 0.05). Based on the results of the Independent Samples T-test, a significant relationship was observed between women of mandatory marriage with total marital burnout and the subscales of somatic, emotional, and psychological burnout (*P* < 0.05). A significant relationship was observed between women with total marital burnout participating in communication skills training courses and the subscales of psychological burnout (*P* < 0.05) (Table 3).

**Table 3:** Relationship between demographic variables with marital burnout variable and its subscales

Variable		Subscales of marital burnout									Total marital burnout		
		Somatic			Emotional			Psychological					
		Mean	SD	*P* value	Mean	SD	*P* value	Mean	SD	*P*	Mean	SD	*P* value
Age *	17–30	17.80	6.48	< 0.001	17.93	7.69	0.440	17.06	6.10	0.252	52.80	18.46	0.003
	31–40	19.36	6.58		18.64	7.47		17.89	5.90		55.90	17.80	
	41–50	20.53	6.17		18.77	7.35		17.39	5.50		56.70	16.41	
	51 <	23.19	7.65		19.17	8.59		18.10	5.79		6.046	19.57	
Husband's Age *	17–30	17.26	6.66	< 0.001	17.32	7.92	0.118	16.44	6.19	0.043	51.04	18.97	< 0.001
	31–40	18.64	6.33		17.38	6.89		1.742	5.61		54.45	16.93	
	41–50	19.71	6.21		1.880	7.96		17.97	6.01		56.48	17.85	
	51 <	22.79	7.25		19.17	8.01		17.87	5.63		59.84	18.24	
Duration of marriage*	1–10	17.69	6.33	< 0.001	17.97	7.63	0.396	17.18	5.90	0.397	52.85	17.89	0.003
	11–20	19.64	6.61		18.97	7.46		18.86	5.92		56.48	17.91	
	21–30	19.70	6.52		18.32	7.43		17.39	5.71		55.42	17.18	
	31–40	22.08	6.58		18.63	8.36		17.63	5.71		58.35	17.37	
	41 <	25.69	8.70		20.49	8.96		19.32	5.31		65.51	22.11	
Occupation**	Housekeeper	19.67	6.65	0.050	18.63	7.69	0.336	17.60	5.83	0.372	55.91	17.90	0.152
	Employee	18.67	6.95		18.08	7.52		17.20	6.04		53.96	18.40	
Husband's Occupation**	Employee	19.23	6.56	0.351	17.73	7.87	0.009	16.87	5.45	0.004	53.84	16.88	0.017
	Self- Employee	19.66	6.82		19.07	5.45		18	6.16		56.73	18.73	
Level of education*	Illiterate	29.50	7.07	< 0.001	26.65	6.73	< 0.001	21.80	5.84	0.063	77.95	19.37	< 0.001
	elementary	20.20	7.32		17.76	8.40		17.18	5.91		55.15	18.68	
	middle school	21.71	6.11		18.37	7.84		17.31	5.53		56.40	16.11	
	High school	19.34	6.75		19.19	7.28		17.91	6.35		56.45	18.79	
	Academic	18.19	6.22		17.74	7.90		17.10	5.33		53.04	16.88	
Husband's education*	Illiterate	24	7.72	< 0.001	20.18	7.84	0.011	18.21	7	< 0.001	62.40	19.31	< 0.001
	elementary	20.67	7.40		18.54	7.59		18.08	6.26		57.30	19.21	
	middle school	20.50	6.95		20.05	8.30		19.09	6.34		59.64	19.20	
	High school	19.45	6.81		18.63	7.33		17.30	5.63		55.39	17.60	
	Academic	18.49	6.21		17.60	7.45		16.87	5.56		52.97	17.09	
Number of children*	does not have	19.53	8.55	< 0.001	22.51	9.60	0.267	20.93	8.07	0.079	62.98	24.11	0.254
	One	18.41	6.37		18.59	7.41		17.48	5.63		54.49	17.31	
	Two	19.47	6.08		18.72	7.45		17.96	5.82		16.15	17.08	
	Three or more	21	7.20		18.51	7.81		17.31	5.84		56.83	18.63	
Relative to spouse**	Relative	19.87	6.77	0.095	18.76	7.76	0.365	17.98	5.87	0.062	56.63	17.96	0.106
	Non-relative	19.10	6.64		18.29	7.56		17.23	5.82		54.63	17.91	
Meet the wife before marriage**	Yes	19.75	6.78	0.230	18.48	7.50	0.984	17.38	5.63	0.480	55.65	17.62	0.835
	No	19.22	.6.68		18.49	7.76		17.65	6.06		55.37	18.36	
Mandatory marriage**	Yes	21.91	5.70	0.008	24.18	8.79	< 0.001	21.02	6.85	< 0.001	67.12	18.79	< 0.001
	No	19.29	6.76		18.18	7.47		17.34	5.77		54.81	17.79	
Marital satisfaction**	Yes	19.16	6.60	< 0.001	17.86	7.10	< 0.001	17.01	5.42	< 0.001	54.04	16.89	< 0.001
	No	23.79	7.09		28.76	8.98		26.03	6.81		78.59	20.29	
Participate in communication skills training courses **	Yes	18.68	6.07	0.050	17.77	7.12	0.096	16.77	5.63	0.031	53.23	16.47	0.029
	No	19.65	6.89		18.73	7.79		17.77	5.94		56.15	18.44	

* One-way ANOVA, ** Independent samples t-test

The results of the Pearson Correlation Coefficient showed that there was a significant correlation between the subscales of somatic, emotional, and psychological burnout and total marital burnout. There was also a significant correlation between the marital burnout subscales (*P* < 0.001) (Table 4). The results of linear regression showed that there was a significant relationship between mandatory marriage, marital satisfaction, marriage duration, and the husband's level of education that coincided with the women's marital burnout. The variables finally predicted 12% of marital burnout variance. It should be noted that marital satisfaction highly effected the prediction of marital burnout (*P* < 0.001) (Table 5).

**Table 4:** Pearson's correlation between the dimensions of marital burnout

Variable	Subscales of marital burnout	Total marital burnout		
	Somatic	Emotional	Psychological	
Somatic	1			
Emotional	0.787*	1		
Psychological	0.920*	0.704*	1	
Total marital burnout	0.865*	0.632*	0.713*	1

* < 0.001

**Table 5:** Results of linear regression analysis in predicting marital burnout

Variable	B	SE	Beta	t	*P* value	Adjusted R Square	F	*P* value
Relative to spouse	− 0.662	1.445	1.018	-0.458	0.647	0.12	12.643	< 0.001
Meet the wife before marriage	− 0.996	1.416	0.027	-0.703	0.482			
Mandatory marriage	− 6.240	2.660	0.077	2.346	0.019			
Marital satisfaction	22.904	2.626	0.290	8.723	0.001			
Participate in communication skills training courses	1.212	1.386	0.028	0.875	0.382			
Education	0.559	0.706	0.034	0.792	0.429			
Husband's education	1.211	0.581	− 0.076	− 2.086	0.037			
Duration of marriage	0.169	0.068	0.110	2.500	0.013			

## Discussion

Based on the results of the present study, there was a significant relationship between marital burnout and age of women, duration of marriage, husbands' job, women's education level, number of children, mandatory marriage, marital satisfaction, and their participation in communication skills training courses. Also, marital satisfaction was one of the effective factors in predicting marital burnout.

The results of the present study showed that total marital burnout and subscales of somatic, emotional, and psychological burnout in women were relatively high. The results of various studies have shown that the rate of marital boredom is higher in women than men [5, 16, 17, 28, 29]. Women have different roles in married life than men, which can be effective in increasing their burnout than men [17, 28]. Housework and domestic responsibilities, take care and upbringing of child/children would be mainly assigned to women, and can be seen not only as a role, but also as a divine duty in the Iranian context [29, 30].

In a study by Alsawalqa, the results showed that there was a significant relationship between the age of women with total marital burnout and the subscale of somatic burnout. This means that with increasing age, the total marital burnout of women and the subscale of somatic burnout increased [28]. According to Ahrari's study, marital burnout also increases significantly with age [29]. In general, with age, people's physical ability and mental capacity gradually decrease. In some women, the rate of depression increases with age, and this seems to be an effective factor in causing couple-burnout [31–34].

In our study, there was a significant relationship between the duration of marriage with total marital burnout, and somatic burnout. This means that with the increasing duration of marriage, the couples’ burnout increased. The results of various studies showed that marital burnout increased with the increasing duration of marriage [28, 29]. Contrary to the results of the present study, the Allendorf's study results in Nepal and the results of Bulgan's study in Turkey showed that marriage satisfaction increases with the duration of marriage [35, 36]. Increasing marriage satisfaction is one of the effective factors in reducing marital burnout [37, 38]. The difference in the results of these two studies with our study could be due to the different cultures of marriage (traditions, expectations, family influences) and the socio-economic status of the couples in different parts of the world.

There was a significant association between the husband's job with marital burnout, the subscales of emotional and psychological burnout in women, and women who’s their husband's job was self-employed showed a higher rate of marital burnout. The results of a study showed that people who work part-time have a lower rate of marital burnout than those who were unemployed or full-time [28]. According to the results, there was a relationship between working hours and marital satisfaction, and increasing working hours causes a person to not be able to spend enough time with his family due to his job responsibilities, and the level of marital satisfaction decreases [39]. The results of other studies have shown that increasing working hours was a higher risk for divorce among couples and harms the couple's romantic relationship [40, 41].

In this study, a significant relationship was observed between the variable of women's education level with total marital burnout, subscales of somatic and emotional burnout, and people who had a lower level of education also had higher marital burnout. Also, women that their husbands had a lower level of education reported higher marital burnout. The results of studies showed that there was a relationship between the level of education and marital burnout and the rate of marital burnout was significantly higher in people who have a lower level of education [17, 42]. In a study conducted by Şendil, there was a relationship between the level of education and marital burnout, and people who had a lower level of education had a higher rate of burnout and lower life satisfaction [43]. Education level is one of the important factors in increasing the quality of marriage. As the level of education increases, the quality of marriage increases, and the rate of marital burnout decreases [44]. Higher education usually leads to more information and skills in various areas of life, especially marriage, and improves interpersonal relationships [45–47].

In the present study, it was found that there was a significant relationship between the number of children and somatic burnout; those who had more children reported higher levels of somatic burnout. The results of various studies showed that there is a relationship between the number of children and marital burnout; with the increase in the number of children, the rate of marital burnout increased [17, 28]. An increase in the number of children of some married couples increased their conflict rate and their quality of marriage decreased [35, 43]. Various study results showed that, as the number of children of the couple increases time for each other decreased, and then their attention to each other decreased causing the eventual increased marital burnout [17, 28]. As the couple had more children the amount of time and energy that they spent with their children increased, which may have eventually lead to the somatic burnout [17, 28].

There was a significant relationship between higher marital burnout and mandatory marriage, the subscales of somatic, emotional, and psychological burnout, and with those who were forced to choose their husbands. The results of Allendorf's study showed that there is a significant relationship between a woman having a husband chosen for her and the quality of marriage, and those women who freely choose their husband and being satisfied with their married life later experiencing a higher quality of marriage [35]. In a study by Imamoğlu, the results showed that people who chose their spouse had more love, more participation in their own married life, fewer problems with their spouse, and ultimately a higher quality of marriage than other couples did [48]. Usually, freedom in choosing a husband (or in traditional marriages, being satisfied with the choice made for them) makes a person choose a spouse who has similar characteristics, behaviors, and traits as themselves, and this fit can help reduce later differences and ultimately increase the quality of the marriage [49].

There was a significant relationship between marital satisfaction with total marital burnout, subscales of somatic, emotional, and psychological burnout, and people who were more satisfied with their lives had lower marital burnout. The results of studies have shown that there was a negative and significant correlation between marital satisfaction and marital burnout and with increased marital satisfaction, marital burnout decreases [37, 38]. Jacob's results showed that people with higher marital satisfaction also had lower marital burnout [50]. Generally, people are satisfied with their marriage when they have a good relationship with their spouse, their marriage has been able to respond to the emotional and psychological needs of them, and in this situation, people feel less burnout [45, 51].

There was a significant relationship between total marital burnout and participation in communication skills training courses, and the subscale of psychological burnout; those who participated in these courses had a lower burnout rate. The results of various studies showed that participation in communication skills training courses reduced stress between a couple, increased the quality of marriage, increased marital satisfaction, and reducing marital burnout [6, 52–54]. Increasing communication skills reduces marital burnout, and this reduction in marital burnout ultimately reduces an emotional divorce between the couple [55]. In communication courses, people learn skills such as conversation skills, empathy, and conflict resolution, which can play an important role in improving the quality of their relationships and in turn they will be able to have better relationships with their spouses [56, 57].

## Limitation and implications

The strength of this study was in the high sample size. One of the limitations of this study was the self-reported completion of information and the lack of accurate monitoring of information. Another limitation which could contribute to marital burnout was that factors such as psychological status (e.g., depression, anxiety), stressful life events, and economic status were not assessed in this study. This study only examined urban women and did not examine the marital burnout status of rural women.

Based on the results of the present study, it is suggested that the following topics on marital burnout be examined in these future studies: The effect of couples’ psychological status (e.g., depression, anxiety), stressful life events, and economic status. It is also suggested that to increase the generalization of the results investigation of couples’ burnout status; of both men and women in urban and rural areas, should be undertaken. Demographic factors set forth in the present study that have a significant relationship with marital burnout could be considered as prerequisites in pre-marital and post-marital training programs. More attention could also be paid to the couples learning effective communication skills in various training programs.

## Conclusion

According to the results of this study, marital satisfaction was one of the effective factors in predicting marital burnout. Due to the important role of women in the family care foundation and in order to ensure and maintain women's health, it can be concluded that it is necessary to pay more attention to this issue. Educational programs and examining the factors that enhance marital satisfaction are needed to prevent and reduce marital burnout in married couples. It is also necessary to hold pre-marriage counseling and education programs to reduce burnout in couples.

## Availability of data and materials

The data sets used and/or analyzed during the current study was available from the corresponding author on reasonable request.
